# Observer aging and long-term avian survey data quality

**DOI:** 10.1002/ece3.1101

**Published:** 2014-05-26

**Authors:** Robert G Farmer, Marty L Leonard, Joanna E Mills Flemming, Sean C Anderson

**Affiliations:** 1 Department of Biology, Dalhousie University1355 Oxford Street, Halifax, Nova Scotia, B3H 4R2, Canada; 2 Department of Mathematics and Statistics, Dalhousie University1355 Oxford Street, Halifax, Nova Scotia, B3H 4R2, Canada; 3 Earth to Ocean Research Group, Department of Biological Sciences, Simon Fraser UniversityBurnaby, British Columbia, V5A 1S6, Canada

**Keywords:** Avian ecology, citizen science, observer error, point counts, population trend estimation

## Abstract

Long-term wildlife monitoring involves collecting time series data, often using the same observers over multiple years. Aging-related changes to these observers may be an important, under-recognized source of error that can bias management decisions. In this study, we used data from two large, independent bird surveys, the Atlas of the Breeding Birds of Ontario (“OBBA”) and the North American Breeding Bird Survey (“BBS”), to test for age-related observer effects in long-term time series of avian presence and abundance. We then considered the effect of such aging phenomena on current population trend estimates. We found significantly fewer detections among older versus younger observers for 13 of 43 OBBA species, and declines in detection as an observer ages for 4 of 6 vocalization groups comprising 59 of 64 BBS species. Consistent with hearing loss influencing this pattern, we also found evidence for increasingly severe detection declines with increasing call frequency among nine high-pitched bird species (OBBA); however, there were also detection declines at other frequencies, suggesting important additional effects of aging, independent of hearing loss. We lastly found subtle, significant relationships between some species' published population trend estimates and (1) their corresponding vocalization frequency (*n* ≥ 22 species) and (2) their estimated declines in detectability among older observers (*n* = 9 high-frequency, monotone species), suggesting that observer aging can negatively bias long-term monitoring data for some species in part through hearing loss effects. We recommend that survey designers and modelers account for observer age where possible.

## Introduction

Wildlife management activities benefit from high-quality, long-term population trend estimates, many of which can be derived from ecological monitoring datasets collected by volunteer observers. These “citizen science” datasets are becoming increasingly popular throughout the world (Dickinson et al. 2010) and collect information on taxa such as birds (Peterjohn 1994), anurans (Blaustein et al. 1994; Lotz and Allen 2007), invertebrates (Kremen et al. 2011), and marine life (Goffredo et al. 2010), among others. Avian datasets are particularly extensive and influential; for instance, data from the annual North American Breeding Bird Survey (“BBS” [1966-present]; Peterjohn 1994) include nearly 50 years of annual visits and influence species-at-risk assessments (Greenberg and Droege 1999; Dunn 2002; Sauer and Link 2011), along with helping to characterize the broad-scale effects of introduced species (Cooper et al. 2007), diseases (LaDeau et al. 2007), and climatic variation (Link and Sauer 2007; Link et al. 2008; Wilson et al. 2011). Although these datasets are a critical resource for understanding long-term ecological patterns, their use of multiple observers over these long terms may introduce a variety of observer errors (Kendall et al. 1996; Griffth et al. 2010).

Population modelers can estimate and correct for detection errors of many forms (Link and Sauer 2002; Royle and Link 2006; Nichols et al. 2009; Miller et al. 2011; but see Campbell and Francis 2011); however, in long-term ecological datasets like the BBS, dynamic, within-observer sources of error are not routinely considered. For example, except for controlling for a first-year “start-up” learning effect (Kendall et al. 1996; Link and Sauer 2002), the current approach to analyzing BBS data taken by the United States Geological Service (e.g., Link and Sauer 2002; Sauer and Link 2011) does not consider length of service (i.e., aging effects) as a relevant covariable.

**Figure 1:**
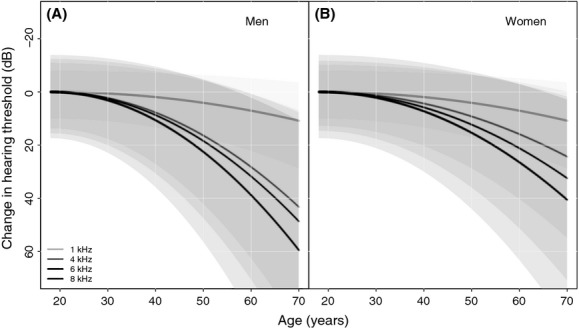
International standard for median expected changes in hearing thresholds at standard pure-tone test frequencies (1, 4, 6, and 8 kHz) among (A) men and (B) women of increasing age. Shaded areas are 95% quantiles. Curves are derived from models specified in International Organization for Standardization (2000).

Nonetheless, aging effects such as hearing loss might play an important role. For instance, we know that in general, the human ability to hear high-frequency sounds – including those produced in birdsong (Mayfield 1966; Emlen and DeJong 1992) – diminishes over time beginning after age 20 for both men and women (International Organization for Standardization 2000; Agrawal et al. 2008; Fig. 1). In addition to age-related hearing loss, many aging people are simultaneously prone to the cumulative effects of noise-induced hearing loss at medium (3–6 kHz) frequencies (Nondahl et al. 2009; Osei-Lah and Yeoh 2010). Furthermore, most avian monitoring data such as BBS records consist predominantly of aural detections (Cyr 1981; Faanes and Bystrak 1981) made over many years by observers over 45 years of age (Fig. S1 in Appendix S1; and see Wiedner and Kerlinger 1990; La Rouche 2001; Downes 2004; Carver 2009). Finally, in spite of many observers having very short (i.e., <2 year) terms of service, the average participation on a BBS route is at least 7 years (Downes 2004), with more than 55% of BBS surveys (unique combinations of observer, route, and year) corresponding to observers serving for 5 years or more (Canadian and US BBS data, 1966–2007, Figure S2 in Appendix S1). If declines in hearing ability as participants age are an important, unrecognized source of missed detection errors, population trend estimates from the BBS and similar datasets could carry an unrecognized bias.

Several studies have hypothesized that aging-related changes to observers – especially hearing ability – might bias current models of bird species counts (e.g., Faanes and Bystrak 1981; Ramsey and Scott 1981; Emlen and DeJong 1992; Simons et al. 2007); however, these have had a limited quantitative scope, or else have not worked with real survey data. For instance, Faanes and Bystrak (1981) considered 15 years' of data comparing relative counts among only 3 BBS observers (routes), showing lower expected counts for hearing-impaired (*n* = 1) versus unimpaired (*n* = 2) observers. Similarly, Ramsey and Scott (1981) measured pure-tone hearing deficits in a large selection of active birders (*n* = 274), but did not consider birdsong specifically, nor how these patterns relate to counts or trend estimates in real data. Emlen and DeJong (1992) observed hearing differences among 2 observers aged 25 and 70, but again only speculated on its impact in real ornithological data using theoretical measures. Using a controlled field setup, Simons et al. (2007) showed substantial underestimation of abundance of the Black-and-white Warbler (*Mniotilta varia*) among 15 observers and argued that age-related hearing loss is probably driving this pattern, but here did not explicitly measure observer age and its association with detection, and only considered this single species in their argument.

To our knowledge, only Link and Sauer (1998) considered observer age and its functional impacts explicitly using a dataset containing more than three observers. In this case, the authors predicted a “43% diminution of counts” for Blue-gray Gnatcatchers (*Polioptila caerulea*) among BBS observers who have conducted surveys for more than 20 years. This “observer senescence effect” was a very limited discussion point in a much broader research paper and was not further explored.

Hence, our understanding of the errors that might result from hearing loss and other aging phenomena in models of long-term survey data is still poor, in spite of their potential to affect population trend estimates and management actions. A problem of this nature is unlikely to disappear, as it is intrinsic to any survey route visited exclusively and repeatedly by a single observer, who must necessarily age over the course of such service. We believe there is a need for future research that explicitly measures any errors resulting from observer aging.

Our goals for this study were to test for the existence and consequences of age-related declines in the detection abilities of long-term bird survey observers, with a focus on hearing loss as a potential mechanism. We used data from two independent volunteer bird survey datasets: the Atlas of the Breeding Birds of Ontario (“OBBA”; Bird Studies Canada et al. 2008) and the BBS (Peterjohn 1994) to measure how bird detection probabilities and expected counts, respectively, tend to change as observers age.

We considered the role of hearing loss by also testing for patterns between observer aging, observer detection ability, and vocalization frequencies of a variety of bird species (Appendix S2). We expected to see the strongest effects for species having vocalization frequencies that corresponded to common age-associated hearing impairments.

We then asked how age-related changes in observer ability might be affecting long-term estimates of population change. First, we tested for a relationship between long-term population trend estimates (derived externally by each of the Canadian Wildlife Service and the US Geological Service using BBS data) and the vocalization frequencies of their corresponding species. We expected that these trend estimates would be more negative in species with vocalization frequencies corresponding to common forms of hearing loss.

Next, we tested for the effect of observer aging more generally on population trend estimates by correlating our own estimates of differences in detection among younger and older observers for specific species (derived earlier) with the same published, long-term population trends. Here, we also expected a negative relationship, where species showing greater declines in detectability among older observers would tend to have more negative population trend estimates.

## Methods

### Calculating and classifying vocalization frequencies

In all analyses, we focused on a group of North American songbirds (warblers, nuthatches, flycatchers) for which we could obtain high-quality vocalization data (Appendix S2). These species form a major proportion of North American breeding bird species (e.g., http://www.aba.org/checklist/), they represent a broad range of vocalization frequencies (Brand 1938), and they are frequently of conservation interest (e.g., Faaborg et al. 2010).

We downloaded audio files of birdsong from the Macaulay Library of the Cornell Laboratory of Ornithology (http://macaulaylibrary.org). We then categorized these species according to their acoustic characteristics by first determining the peak (i.e., dominant) frequencies for the vocalizations of each species following Emlen and DeJong (1992; see Appendix S3 for details). We then used these peak frequency values to assign species into one of four vocalization frequency groups corresponding to thresholds of 3, 6, and 7 kHz. These thresholds help to isolate frequency ranges more likely to be affected by hearing loss phenomena, where age-related hearing loss tends to occur in an increasing fashion above 6 kHz (International Organization for Standardization 2000; Gates and Mills 2005), and noise-induced hearing loss tends to occur in a “notched” range between 3 and 6 kHz (Nondahl et al. 2009). Accordingly, species were considered to have “low” (<3 kHz), “notch” (≥3 and <6 kHz), “medium” (≥6 and <7 kHz), and “high” (≥7 kHz) vocalizations.

“Peak frequency” is most representative of a particular vocalization if the overall vocalization broadcasts a very narrow range of sounds (frequencies) (Fig. S3 in Appendix S1; Ramsey and Scott 1981). By comparison, it is less representative of vocalizations incorporating many different musical notes (frequencies). To account for this difference and focus on the former type, we thus further classified vocalizations as being either “monotone” or “heterogeneous” according to the range of frequencies found in each power spectrum (standard deviation of power values). We defined “monotone” vocalizations as those vocalizations with power spectra having a standard deviation less than or equal to the median value among a group of 94 species initially considered; all other vocalizations having more variable power spectra were defined as being acoustically “heterogeneous”. Using these heterogeneity classes, we thus expanded the existing four vocalization groups discussed above into eight (i.e., “Low Monotone,” “Low Heterogeneous,” “Notch Monotone,” “Notch Heterogeneous,” “Medium Monotone,” “Medium Heterogeneous,” “High Monotone,” and “High Heterogeneous”; Appendix S2). We expected that any relationships between species detections and vocalization frequencies resulting from frequency-specific hearing loss phenomena would be stronger among monotone species.

This simple classification method did not recognize cases where bird vocalizations featured a wide range of frequencies broadcast over a very short time interval (e.g., Least Flycatcher [*Empidonax minimus*]) – and so which appear subjectively monotone to the human ear in spite of their having a heterogeneous power spectrum. However, this error did not risk the inclusion of subjectively heterogeneous species in the monotone groups – a more serious error because we were largely concerned with patterns among monotone species only – and so this error was a conservative one.

### Determining observer-age-related differences in detection probabilities and counts

#### Age-related differences in detection probabilities

To estimate the difference in detection probability between older and younger observers, we used data from 43 species surveyed as part of the OBBA that had at least 100 detection records in total (Appendix S2), and for which we were able to determine peak vocalization frequencies. The OBBA is a volunteer survey that divides the entire land area of the Canadian province of Ontario into a grid of 3324 10 × 10 km squares. During two 5-year periods (“first atlas”: 1981–1985; “second atlas”: 2001–2005), one to several volunteers per square conducted area searches for breeding evidence of bird species during the spring and summer months, with a minimum effort of 20 party-hours per square.

Working with atlas squares sampled between 2001 and 2005 (“second atlas”) during at least two separate years by one or more observers, we inferred species detections as occurring when an atlasser reported any evidence of a given species in a given atlas square. Conversely, we inferred nondetection for a given atlasser and species by determining all squares visited by an atlasser and assigning zeroes (“no detection”) to all species that were not reported there (Kéry et al. 2010).

We used publicly available data sources, including OBBA results web pages and field naturalist groups' newsletter reports, to subjectively determine the approximate ages (under 40, 40–50, or over 50) during the midpoint of the second atlas (2003) for 626 of 1230 atlassers (demographic data were not available for many observers). In the subsequent analyses, we excluded records for atlassers we believed to be aged between 40 and 50 years to increase the chance that “younger” and “older” individuals were correctly classified. Although our primary interest was in measuring age-related patterns of detection ability, we also recognized that gender could have an influence, because men tend to lose their high-frequency hearing sooner than women ( Fig. 1), and so we recorded gender as well. We also corrected for observer effort, both by excluding records with zero-effort values (presumably a problem of missing effort data) and by modeling species detectability with effort as a covariable.

We could not explicitly distinguish between casual, area-restricted “backyard” observations and more-dedicated, extensive field searches within a given atlas square, the former of which might be more often recorded by older, less mobile bird-watchers. Any such relationship, if widespread in the data, could confound age-related differences in observer detection abilities with age-related differences in observer mobility. However, we were unlikely to successfully determine an observer's age and hence, include that observer's data, unless he or she was generally active in the bird-watching community, sufficient to warrant publishing his or her name and photograph in a field naturalist group's newsletter or similar publication. In our experience, active field naturalist group participation implies an ability and a preference to visit sites further afield than a backyard. Hence, we suspect that this potential confounding influence is not widespread in the data used here.

To model the effects of observer age on detection probability, we constructed Bayesian hierarchical occupancy models of detection data for each species (Royle and Kéry 2007; Royle and Dorazio 2009) in WinBUGS 1.4.3 (Lunn et al. 2009) and R 2.13.0 (R Development Core Team 2011) using the R package *arm* (Gelman et al. 2010) on a PC running Windows 7. The model structure, following Royle and Kéry (2007), consisted of two parts: (1) a 0–1 occupancy variable for the 2001–2005 survey, estimated as a Bernoulli process using a latent logistic probability function of 1981–1985 detection (an assumed positive predictor variable), and (2) a logistic detection probability model conditional on the occupancy model, expressed as a function of (2001–2005) surveyor effort, age (over 50 vs. under 40), and gender. The occupancy (1 or 0) and detection (probability) components were multiplied, assuming independence, to obtain a binomial detection function that was fit to the data using minimally informative priors.

Consistent with a fundamental assumption of OBBA design (necessary to compare patterns of occupancy among separate atlases, which are conducted every 20 years), we assumed that occupancy did not change for a given atlas square during each of the 5 years of an atlas sampling period, and so treated each atlas square as a sampling unit and each sampling year as a within-observer replicate. We did not account for the presence of any false-positive detections in the data (e.g., McClintock et al. 2010b).

In the models, we used up to 350 observers (depending on the species being modeled) from two age groups: observers under 40 (18 women, 65 men) and observers over 50 (64 women, 203 men), and expected that the older cohort would have functionally reduced detection abilities compared with the younger one, on average ( Fig. 1). We did not model any observer characteristics from the first atlas (no observer information was available), but assumed that apparent occupancy by a species during this older survey (1981–1985) increased the prior probability of detection in the second atlas (our principal dataset). Specific formulations of the occupancy models and priors used are discussed in Appendix S4.

“*β*_2_”, an observer age parameter (see equation 5 in Appendix S4) corresponds to the difference in detection probability on the logistic scale between observers older than 50 and younger than 40 for a given species. Negative values imply a decrease in detection ability among older observers. We noted which *β*_2_s were negative, and later related them to bird vocalization frequencies.

We also tested for the sensitivity of the values of *β*_2_ to the inclusion of observers of borderline age (i.e., ages 40–50). We refit the occupancy models as described above, except here using data from observers of all ages (while retaining the age-50 cutoff for “old” vs. “young”), and then compared matched pairs of these new *β*_2_ estimates to their earlier estimates. If there were important, cumulative differences in detection ability between “old” and “young” observers, we expected to see a smaller overall difference in detection ability between the under-50 and over-50 cohorts, compared with the differences previously measured between the under-40 and over-50 cohorts.

#### Age-related differences in counts

Next, we determined how bird counts varied with increasing observer age on the BBS. Like the OBBA, the BBS is a multiyear, omnibus bird survey, conducted by skilled volunteers during the breeding season. In contrast to the OBBA, most BBS survey stops are not replicated within survey periods, and a single observer usually collects all data for a given location each year at a single point in time. Locations consist of a set of permanent, 39.4-km road transects (“routes”), which are divided into 50 stops placed at regular (∼800 m) intervals. Most BBS routes are sited randomly within North American physiographic subregions (“strata”; e.g., “Sierra Nevada”; “St. Lawrence River Plain”) and within degree blocks of latitude and longitude, and so have a nested random structure. The delineation and rationale behind the physiographic strata is outlined in Sauer et al. (2003). Survey routes continue to be added to the BBS as a whole; the oldest routes have been monitored annually since 1966.

In the raw BBS count dataset, observers are assigned a unique identification number, which persists throughout their years of service. We used these identification numbers to determine a measure of “minimum observer age,” defined as the number of years since the first year an observer served on any BBS route (sensu Faanes and Bystrak 1981). Within observers, minimum observer age is correlated with actual observer age – our latent variable-of-interest – by definition. This measure is less precise than true age, however, for simplicity, we refer to “minimum” observer ages, which range from 1 to 39 in the data, as “observer ages”.

We omitted data from the early years of the BBS and instead used data collected in Canada and the USA between 1970 and 2007 by single observers under suitable weather conditions. These omissions avoided potential problems with low observer quality in the early years of the survey (e.g., Sauer et al. 1994), as well as problems with “anomalous results” with early data from Canadian survey routes (see http://ec.gc.ca/reom-mbs/default.asp?lang=En&n=E8974122-1). Observer ages were calculated using the original, complete dataset, which began in 1966. Because raw BBS data do not include zero counts for any species, we added relevant zeros in the same manner as was done with the OBBA dataset, after Kéry et al. (2010).

As a part of this analysis, we needed to control for real changes in population abundance occurring alongside changes in observers' detection ability. This required replicated time series data for each location, which do not exist. We thus controlled for population change at a broader spatial resolution than the individual survey route – here using physiographic strata – and used counts at the individual survey routes as replicates. We required at least three separate observers to be associated with a given stratum before it was included in the analysis. Similarly, to ensure that the age and population effects under study were not confounded, we required that the pooled ages of all observers and the calendar year were not correlated by more than 0.7 (Pearson correlation) for each species and stratum analyzed. We also worked exclusively with observer–route time series surveyed continuously for 10 years or longer, both to minimize errors that could result from any gaps in temporal coverage (e.g., Sauer et al. 1994) and to capture age-related changes in detection ability.

Volunteer BBS observers often perform worse during their first year on a survey route compared with later years; this phenomenon can inflate population trend estimates if the first year of data is included (Kendall et al. 1996). To avoid confounding this pattern with hearing loss phenomena, we excluded the first years' datapoints (mean 6.1% of records per species) for each observer–route combination. Final datasets for each of 64 species meeting these requirements (and for which we had vocalization frequency data; Appendix S2) ranged in size from 37 (Lucy's Warbler [*Oreothlypis luciae*]) to 6692 (Common Yellowthroat [*Geothlypis trichas*]) route years of data, with a median 1077 records.

We used overdispersed Poisson generalized additive mixed models (“GAMMs”; Wood 2006) in R package *gamm4* (Wood 2011) to model the nonlinear changes to BBS counts with observer age. As an extension of generalized additive models (“GAMs”), GAMMs are a nonlinear modeling approach, which make few assumptions about the shape of a relationship between two variables (i.e., observer age and estimated count). Using its parent package *mgcv* (Wood 2006), these models optimize the amount of “wiggliness” using internal cross-validation algorithms (Wood 2006), and their *P*-values correspond to the null hypothesis of no linear nor nonlinear relationship in the data. By design, such smooth functions are intended to be visually inspected by the modeler; summary statistics alone are inadequate to describe the modeled patterns.

Compared with GAMs, GAMMs incorporate additional random-effects structures to account for group-specific deviations from overall means (“random intercepts”) and from overall trends (“random slopes”). Hence, these models are suitable for the hierarchical structure of BBS data. Here, in modeling the relationship between observer age and bird counts, we controlled for among-observer effects using random intercepts and for continuous changes in species counts with calendar year within physiographic strata (i.e., “population” changes) using smooth functions.

Using pooled data from predictions made in this first set of models, we then built a second set of GAMMs – one for each of the vocalization frequency and heterogeneity groups defined earlier – describing the expected proportional changes to BBS counts with increasing observer age, generalized among groups of species. Finer details of this modeling process are outlined in Appendix S5.

### The role of hearing loss

#### Age-related differences in detection probabilities

We tested whether hearing loss might be related to age-related changes to detection ability using additive models (“GAMs”; Wood 2006) built using the R package *mgcv* (Wood 2006). In these models, we predicted the 43 species-specific estimates of *β*_2_, calculated in the earlier analysis of OBBA data, as a function of the peak vocalization frequencies of each corresponding bird species. If age-related hearing loss is an important mechanism leading to age-related detection declines, we expected the *β*_2_ values to be more negative with increasing vocalization frequency. More negative values in the “notched” region would similarly correspond to an influence of noise-induced hearing loss.

We considered relationships between *β*_2_ and vocalization frequency for the monotone and heterogeneous vocalizations separately and weighted the datapoints according to the inverse of the variance of their posterior distributions (i.e., their uncertainty; estimated earlier by WinBUGS). We expected to see more pronounced patterns for the “monotone” species groups, because in this case, the peak vocalization frequency more closely corresponds to the principal frequency broadcast to an observer. We conducted these analyses separately from the initial hierarchical models because, to our knowledge, there is no existing, validated option for constructing GAMs in WinBUGS.

#### Age-related differences in counts

The existing analysis of BBS count data implicitly tested for the role of hearing loss by grouping the results by vocalization frequency and heterogeneity. We expected to see more pronounced count declines over time among the higher-frequency and notched monotone vocalization groups.

### Long-term population trend estimates

We lastly determined whether age-related observer effects might have influenced existing broad-scale population trend estimates. Here, we considered Canada-wide population trend statistics produced by both the US Geological Service (“USGS”; http://www.mbr-pwrc.usgs.gov/cgi-bin/atlasa09.pl?CAN&2&09) and by the Canadian Wildlife Service (“CWS”; http://www.cws-scf.ec.gc.ca/mgbc/trends/index.cfm?lang=e&go=info). Both sets of trends are calculated by their respective agencies using area-weighted, Poisson-modeled BBS count data, where estimated “trend” values correspond to the estimated exponential rate of change in a population from the beginning to the end of the survey period modeled. However, fine details of these strategies are not equivalent. Thomas and Martin (1996) showed that agency-specific differences in analysis methods (i.e., different geographic weighting schemes) can lead to important differences in trend magnitude and significance. Current trend estimation strategies have improved since 1996 among both agencies, but remain divergent for other reasons (C. Francis, pers. comm.). Here, we wanted to determine whether there was an agency-independent (i.e., common) effect of vocalization frequency among each set of trends, and so considered both.

If hearing loss is an important mechanism for age-related detection declines, we expected to see greater estimated population declines among species with vocalization frequencies associated with hearing loss. To test for such a pattern, we built GAMs relating each of the USGS and CWS population trends with species vocalization frequency, specifying separate thin-plate regression spline smoothers for monotone and for heterogeneous vocalizations. The USGS dataset supplied 95% credible intervals for each initial trend estimate; consequently, we treated the width of these intervals as a measure of error and weighted datapoints according to their inverses. Similarly, we used the supplied number of BBS routes incorporated into each CWS trend prediction as a corresponding weight in the CWS GAM. We used population trend estimates that spanned the longest available time span in each case, which was from 1966 to 2009 for the USGS trends (*n* = 50 warbler, flycatcher, and nuthatch species for which we had vocalization frequency data; Appendix S2), and from between 1970 and 1973 to 2009 for the CWS trends (*n* = 52 species; Appendix S2). We excluded one CWS trend (Bohemian Waxwing [*Bombycilla garrulus*]), which was valid only for 1986 to 2009.

We then asked whether observer aging in general might be adding error to these trends by comparing our estimates of observer-age-related differences in detection probability (i.e., *β*_2_ in the OBBA data) to the mean USGS and CWS population trend estimate data (derived largely from BBS data; Sauer and Link 2011) using Pearson correlations. To avoid correlating statistical noise, we considered only those species which already showed a significant relationship between detection probability and vocalization frequency, namely medium- and high-frequency monotone birds (*n* = 9 species; see Results).

## Results

### Determining observer-age-related differences in detection probabilities and counts

#### Age-related differences in detection probabilities

**Figure 2:**
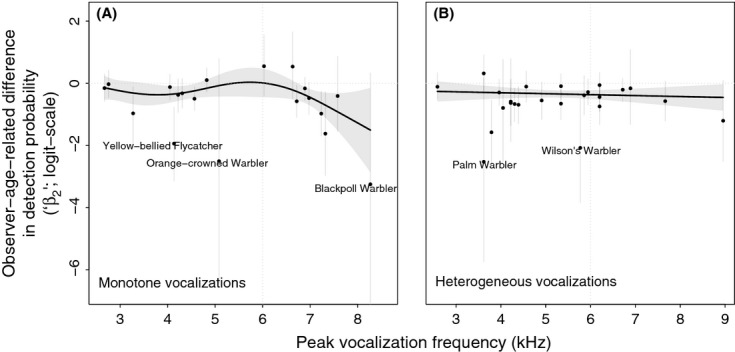
Logit-scale difference in species detection probability between an observer over age 50 and an observer under age 40 (“*β*_2_” coefficients), determined from species-specific hierarchical occupancy models of data from the Atlas of the Breeding Birds of Ontario, as a function of each species' peak vocalization frequency, and grouped by vocalization variability (“Monotone” [A] and “Heterogeneous” [B]). Smoothed curves are GAM fits, weighted by the inverse of the variance of each datapoint (uncertainty displayed as 95% credible interval lines here), plus the model intercept. Shaded areas are 95% pointwise confidence bands about the smooth term and the model intercept. Dotted reference lines are plotted at *y* = 0 (no difference in detection between younger and older observers) and at 6 kHz (the threshold for “medium” frequencies used in this research).

For each species surveyed on the OBBA, the *β*_2_ estimates measured expected differences in detection abilities between the older and younger observer cohorts. Negative values indicated that a species is less likely to be detected by an older observer compared with a younger observer. Their average among all 43 species was negative (mean −0.66, 95% bootstrapped quantiles: [−3.48, 0.723]; median −0.48; Fig. 2). These values were not normally distributed and were left-skewed. When modeled in the GAM, the corresponding intercept term, which indicates a central tendency, was significantly negative (*P* < 0.001).

On a species-specific basis, 13 of the 43 species considered (30%; BAWW, BBWA, BTNW, COYE, CSWA, GCKI, NAWA, OSFL, OVEN, RCKI, WIWA, YBFL, YRWA; see Appendix S2 for full names) showed “significant” declines in detectability between younger and older OBBA observers (i.e., 95% Bayesian credible intervals of *β*_2_ coefficients did not contain zero). None of the *β*_2_ values for the 43 species was significantly greater than zero. Gender had a less important influence on detection probability; in this case, the mean effect was closer to zero (0.17 ± 0.59 [SD]), and seven of the 43 (16%) species showed “significant” effects of being male on detectability. Contrary to our physiological expectations, each of these significant effects of being male was greater than zero, indicating a positive effect of male gender on detection.

**Figure 3:**
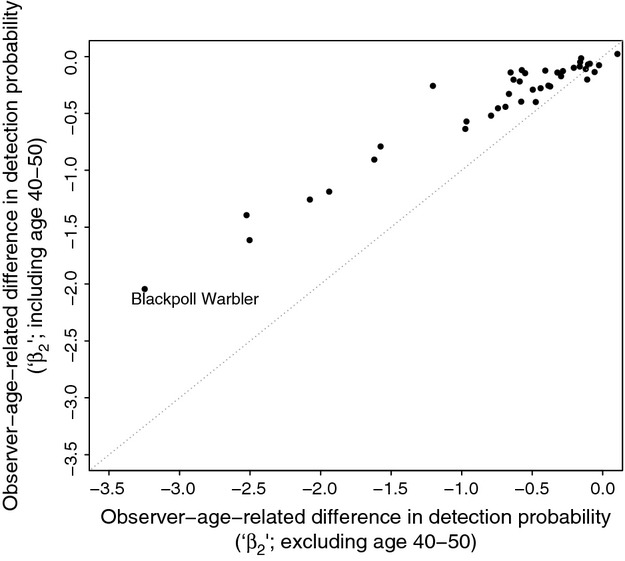
Sensitivity of observer-age-related differences in species detection probabilities (“*β*_2_”) to the age disparity between the old and young age-groups in the modeled data. Coefficients were generated from identical occupancy models using data that either lacked the observers aged 40–50 as part of its “young” category (principal modeling approach; *x*-axis) or included them (*y*-axis). The dotted reference line has a slope of 1. When the additional group of middle-aged (40–50) observers are included in the “under-50” category (*y*-axis), the observer-age-related differences in species detectability are smaller.

The sensitivity analysis tested how including observers aged 40–50 in the “younger” age-group (which formerly contained only observers under 40) affected the relative difference in detection ability between “younger” observers and those over 50 (“older” observers). When observers between ages 40 and 50 were included in the models as “younger” participants, the negative effect diminished in magnitude ( Fig. 3). In other words, the negative effect of older observer age on bird detection probabilities is greater with increasing age disparity.

#### Age-related differences in counts

**Figure 4:**
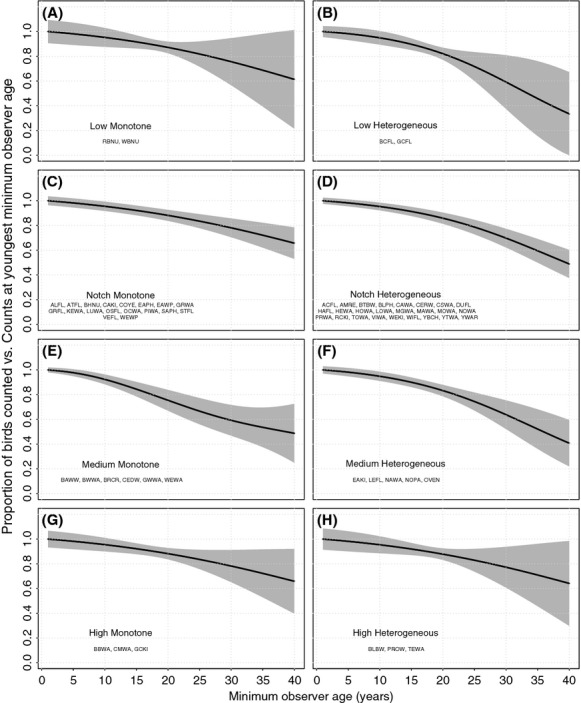
Estimated proportional changes in BBS counts with increasing minimum observer age, relative to the count during the first year of BBS service, grouped by species vocalization groups (Low Monotone [A], Low Heterogeneous [B], Notch Monotone [C], Notch Heterogeneous [D], Medium Monotone [E], Medium Heterogeneous [F], High Monotone [G], High Heterogeneous [H]). Vocalization groups reflect the peak frequency of a typical set of vocalizations for that species, and whether these vocalizations tend to feature a single sound (“Monotone”) or a highly variable set of sounds (“Heterogeneous”). Standard abbreviations for species names (Klimkiewicz and Robbins 1978) belonging to each vocalization group are listed on each panel (also see Appendix S2). Shaded areas are 95% pointwise confidence bands.

Model-estimated BBS counts declined monotonically and significantly (GAMM smooth term *P* < 0.05; Fig. 4) over 39 years of increasing observer age for all vocalization frequency groups except Low Monotone (*n* = 2 species; *P* = 0.111) and High Heterogeneous (*n* = 3 species; *P* = 0.085). Those vocalization groups showing significant changes incorporated data from 59 grouped species ( Fig. 4).

### The role of hearing loss

#### Age-related differences in detection probabilities

Among both the monotone and heterogeneous species groups in the OBBA data, age-related detectability differences were not significantly related to vocalization frequency (GAM smooth terms: *P* = 0.297 [monotone], *P* = 0.597 [heterogeneous]; see Fig. 2 to interpret the pattern). However, the shape of the curve for monotone species suggested focal deficits in detectability between 3 and 6 kHz (“notch” frequencies) and beyond a threshold of approximately 6 kHz (“medium” and “high” frequencies; Fig. 2A). To test for observer-age-related differences in detectability above the latter (≥6 kHz) frequency threshold, we built a *post hoc* linear model predicting observer-age-related differences in detectability as a function of peak vocalization frequency, using only those species with monotone vocalizations above 6 kHz (GWWA, BWWA, BAWW, CEDW, BRCR, GCKI, BBWA, CMWA, BLPW; see Appendix S2), also weighted for uncertainty in the detectability estimates. This model showed a significant linear decline (*P* = 0.034, *n* = 9 species).

#### Age-related differences in counts

Among the significant declines estimated from the BBS data, the greatest absolute changes in expected counts were among the low-frequency, heterogeneous species (BCFL, GCFL, Appendix S2), which decreased by 66.5% over the 39 years sampled, and the medium-frequency, heterogeneous species (EAKI, LEFL, NAWA, NOPA, OVEN; Appendix S2), which decreased by 59.2%. The smallest significant changes in counts were declines of 34.1% among high-frequency monotone birds (BBWA, CMWA, GCKI, Appendix S2) and 34.3% among notch-frequency monotone birds (*n* = 18 species; Appendix S2) over that same age range ( Fig. 4). The increasing uncertainty at the upper range of observer ages ( Fig. 4) reflects the smaller sample sizes in this area.

### Long-term population trend estimates

**Figure 5:**
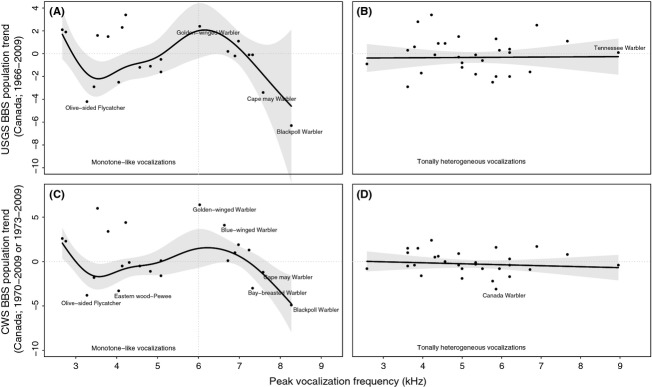
Additive models of Canada-wide population trends calculated by the United States Geological Service (1966–2009; panels A and B), and by the Canadian Wildlife Service (1970 to 2009; panels C and D), as a function of each species' peak vocalization frequency (pitch), modeled separately for species with largely single-frequency vocalizations (“Monotone”; panels A and C; *P* < 0.05 in both cases) and for species with highly variable vocalization frequencies (“Heterogeneous”; panels B and D; *P* > 0.05 in both cases). The models weighted each datapoint (trend estimate) according to a rough estimate of its reliability (95% credible intervals [USGS], and number of associated survey routes [CWS]). Shaded areas are 95% pointwise confidence bands.

There were significant nonlinear relationships between monotone vocalization frequencies and long-term, Canada-wide population trends for each of the USGS (GAM *P* = 0.048, *n* = 22 species; Fig. 5A) and CWS datasets (*P* = 0.008, *n* = 23 species; Fig. 5C). Here, population trends were visually more negative among species with increasing “medium” and “high” (≥6 kHz) peak vocalization frequencies, and at the midpoint of the “notched” range (3–6 kHz). By contrast, there were no significant relationships between heterogeneous vocalization frequencies and population trends (USGS *P* = 0.928, *n* = 28; CWS *P* = 0.568, *n* = 29; Fig. 5B and D). Excluding the potentially outlying Blackpoll Warbler (*Dendroica striata*) did not affect the significance of the monotone USGS relationship (*P* = 0.049), but removed it for the monotone CWS relationship (*P* = 0.064).

**Figure 6:**
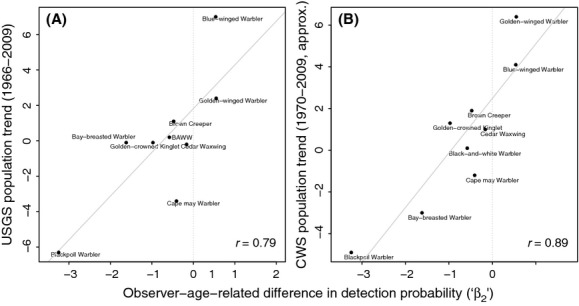
Observer-age-related differences in species detection probabilities (“*β*_2_” coefficients) estimated by the OBBA occupancy models plotted against (A) USGS and (B) CWS long-term population trend estimates for Canada, for those species common to the two analyses and having monotone vocalizations of medium (≥6 kHz) or high (≥7 kHz) frequencies. Solid lines correspond to linear regression fits which ignore uncertainty in the detection probability (*x*-axis) values. Both regression slopes are significantly (*P* < 0.05) different from zero. “BAWW” is the Black-and-white Warbler (*Mniotilta varia*). This figure combines information from Figures 2 and 5.

The above patterns (vocalization frequencies versus population trends; Fig. 5) were also visually similar to those we observed between vocalization frequencies and age-related changes to detection probability (i.e., Fig. 2). The relationship between population trends and estimated detection probability changes for medium- and high-frequency monotone species was significant using both the USGS data (Pearson correlations: *r* = 0.79, *P* = 0.012; *n* = 9; Fig. 6A) and the CWS data (*r* = 0.89, *P* = 0.001; *n* = 9; Fig. 6B). Excluding the Blackpoll Warbler here removed the significance of the USGS relationship (*r* = 0.58, *P* = 0.128), but did not affect the CWS relationship (*r* = 0.86, *P* = 0.006).

## Discussion

Using data from both detection–nondetection (OBBA) and point-count (BBS) surveys to model detection probability and expected counts, respectively, we found evidence of age-related declines in bird detection abilities among volunteer observers. While our results tend to vary among species and species groups, on the whole, they seem to reveal a prevalent, if subtle, phenomenon.

Using the OBBA dataset, we found that observers over age 50 had significantly lower detection probabilities compared with observers under age 40 for 13 of the 43 bird species studied ( Fig. 2). The sensitivity analysis also showed a strengthening pattern of age-related declines among species when the difference in age between “old” and “young” observers increased ( Fig. 3). Our analysis of BBS data, which measured aggregated patterns among species vocalization groups, also showed significant declines in expected BBS counts with increasing observer age among 4 of 6 species groups comprising 59 species ( Fig. 4).

Although we found evidence that hearing loss may be a mechanism for the observed age-related declines in observer detection ability, a broader subset of species than we expected was affected, suggesting that other mechanisms are probably also at play. For instance, while we expected the detectability of the medium- and high-frequency monotone species to decline with age as a result of age-related hearing loss, in the BBS dataset, the magnitude of these declines was shallower than in groups of lower-frequency and heterogeneously calling species, which we predicted would show less pronounced declines, if any (i.e., Fig. 4C and G; 34.1% [High Monotone] vs. 66.5% [Low Heterogeneous]). Similarly, while we observed a significant linear decline in detectability with increasing vocalization frequency among the medium- and high-frequency monotone birds in the OBBA data ( Fig. 2), there were also detection declines at other call frequencies.

Normal aging can involve impairments in memory, cognitive speed, and vision (Morris and McManus 1991). Alongside hearing impairments, these intrinsic observer factors might each contribute to missed detections independent of bird vocalization frequencies, for instance, by limiting one's abilities to simultaneously detect and transcribe species calls, recognize multiple, overlapping species calls, and identify nonvocal, cryptic species by eye (i.e., variable species “conspicuousness”; Stewart 1954; Alldredge et al. 2007) This could explain the presence of more widespread age-related detection declines among the different vocalization frequency groups.

Many older bird watchers are also likely to be more experienced and consequently more adept at detecting a wide range of rare and common species. For effectively sampling such a range of species, this experience advantage may outweigh some physiological deficiencies (Ramsey and Scott 1981) and may also contribute to the more mixed picture of age-related detection declines seen here. Future controlled experiments using observers of known hearing thresholds, ages, and skill levels, with exposure to a variety of bird vocalizations of known audiological characteristics, would help to distinguish the relative importance of hearing, aging, species behavior, and skill effects.

If this observer error is present in the OBBA, BBS and similar datasets, subtly or not, the important follow-up question is whether it is affecting or has affected current and past population trend estimates. Here, we found indirect evidence of such an error in that recent, published trend estimates tended to be lower both as monotone vocalization frequencies above 6 kHz increased, and in the midpoint of the “notched” frequency range (3–6 kHz; Fig. 5), both ranges of which might be affected by aging-related hearing loss. Among the nine species with high-frequency monotone vocalizations, the population declines predicted by each of the USGS and CWS were significantly correlated with our estimated detection declines ( Fig. 6). However, in this relationship, there was an inconsistent effect of excluding the Blackpoll Warbler (*Dendroica striata*), which had both a very low population trend estimate and a very high monotone vocalization frequency. Excluding the Blackpoll removed the significance of the CWS relationship and suggests that, in general, these errors may again be subtle ones.

The majority of BBS observers participate for less than 5 years, and as a consequence, one might conclude the problem of aging observers is not a serious concern for BBS data quality. We believe that the modal participation length is less important to data quality than is the average “age” of a given data record. In other words, while the majority of observers might participate for relatively few years, most of the BBS data are generated by longer-serving observers. As we report in the Introduction, our analysis of North American BBS data shows that more than 55% of data records correspond to an observer having already served at least 5 years on the BBS (Fig. S2B). In light of this factor and of the results of this research, we believe that the problem of observer aging cannot be safely ignored.

Our study of age-related declines in BBS counts was consistent with the results from the single, previous study of this nature (Link and Sauer 1998). Whereas Link and Sauer (1998) estimated a 43% decline in Blue-gray Gnatcatcher counts among observers after 20 years, we estimated declines ranging from 34% to 67% of the original counts of 59 species considered collectively over 39 years ( Fig. 4). Using our approach to classifying vocalizations by peak frequency, the gnatcatcher – a species for which we did not have sufficient data to analyze – would fall into the Notch Heterogeneous category, for which we estimated a 14.1% decline in counts over 20 years. The smaller value predicted here may result from the large number of species (*n* = 25) incorporated into this latter average. In our opinion, either value is large enough to be concerning.

GAM- and GAMM-based methods are relatively new to ecology (e.g., Fewster et al. 2000; Clarke et al. 2003; Flemming et al. 2010), but we have shown their usefulness correcting for continuous, nonlinear factors such as the changes in detection ability with observer age. Implementing changes to survey data collection protocols could both facilitate and complement a GAMM-based modeling approach. For instance, directly collecting observer ages and quantifying their hearing ability before a survey would improve model precision. Emlen and DeJong (1992) also suggest that administrators recommend the use of hearing aids when hearing tests results show deficiencies. However, there may be legal barriers to collecting and storing these personal data.

Universal double-observer methods with younger partners (e.g., Nichols et al. 2000; Alldredge et al. 2006; but see Fitzpatrick et al. 2009), although perhaps impractical given the limited number of available skilled participants, might also help to account for this source of error. A long-term solution, once the appropriate technologies become sufficiently practical, might involve collecting field recordings using volunteers, and then interpreting their sounds using experts at a central office, rather than relying upon on-site classifications (e.g., Campbell and Francis 2011). Any such protocol changes should aim to be consistent throughout the survey as a whole, they must be cost effective, and ideally, they should not compromise the long-term integrity of the overall time series (e.g., Freeman et al. 2007).

In both analyses, we controlled for many suspected observer-specific confounders by excluding data. For instance, we excluded observers aged 40–50 in the analysis of OBBA data to ensure reliable separation between “young” and “old” groups, thus improving the relative accuracy of our age classifications. We also required a minimum of 10 years of service on the BBS for an observer's data to be included, here to increase the likelihood that aging effects occur for all observers, so that we might measure them. While these conservative approaches were appropriate for precisely determining the nature of observer aging effects, they limit the quality of the real (simultaneous) population trends that can be inferred (Link and Sauer 1997). Future analyses should explore the sensitivity of the observer- and population-specific patterns we observed here to increasingly relaxed data subsetting rules.

We believe that asking older or noise-exposed observers who are at risk of detection errors to consider the possibility of any age-related impairments is itself an important step forward: As with any gradual physiological change, observers over age 50 may not recognize a growing, but significant personal impairment (A. G. Horn, pers. comm.), and awareness of this fact alone may lead to an increased degree of self-selection in terms of opting out of surveys. For instance, 75% of a sample of 253 Audubon Christmas Bird Count observers have indicated a desire to remove themselves from survey duties if such an impairment was recognized (Downes 2004).

In general, our study adds to the growing body of literature demonstrating systematic, long-term changes in BBS survey conditions (e.g., Betts et al. 2007; Griffth et al. 2010) that must be controlled for when estimating measures of population change. We have shown that observer age can be a significant source of error and have suggested how surveys might control for its effects. While this research focused on bird observations in particular, its implications are generalizable to other auditory wildlife surveys, for instance, anuran call counts (e.g., McClintock et al. 2010a). We hope that this research leads to improvements in long-term population trajectory inferences, while preserving the invaluable contributions made by volunteers to worldwide ecological monitoring.

## Supporting Information

**Appendix S1.** Supplementary figures. **Figure S1.** Age distributions among bird surveyor gender cohorts. **Figure S2.** Measures of BBS observer age by (A) unique observer and (B) unique survey (unique combinations of observer, survey route and year), Canada and USA, 1966–2007. **Figure S3.** Examples of audiological power spectra corresponding to monotone (Blackpoll Warbler [*Dendroica striata*]) and heterogeneous (Tennessee Warbler [*Oreothlypis peregrina*]) vocalizations. **Appendix S2.** Table of species used in the various hearing-loss analyses. **Appendix S3.** Vocalization heterogeneity. **Appendix S4.** Hierarchical occupancy model structure. **Appendix S5.** Detailed methods for modeling changes in BBS count data with increasing observer age.

**Data S1.** WinBUGS and R code for building occupancy models and GAMMs, respectively, plus functions to generate testing datasets.
