# Supplementary material for: Observer aging and long-term avian survey data quality
Source: Ecol Evol. 2014 May 26;4(12):2563–76. doi: 10.1002/ece3.1101 (PMC4203298; doi:10.1002/ece3.1101)
Supplement: Supplementary file 1 [file ece30004-2563-sd1.pdf]

Supporting Information (Appendices)

Appendix S1. Supplementary figures

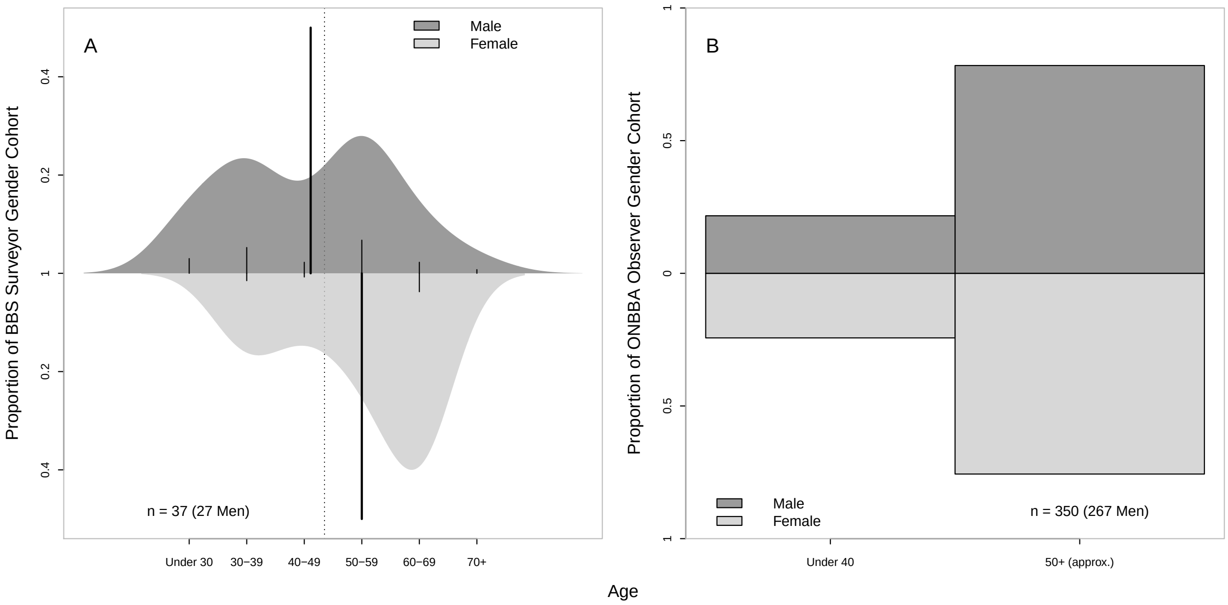

Figure S1. Age distributions among bird surveyor gender cohorts. Panel A shows a beanplot (Kampstra 2008) of the distribution of age ranges among a small sample of BBS observers, based upon demographic information collected by an unrelated internet-based survey of birdwatcher observer effects (Farmer et al. 2012). Tick mark lengths correspond to observer abundance at each age range; the dotted line is the overall mean, solid lines are group means. Panel B shows a barplot of the genders and estimated ages of those OBBA observers determined for the current study.

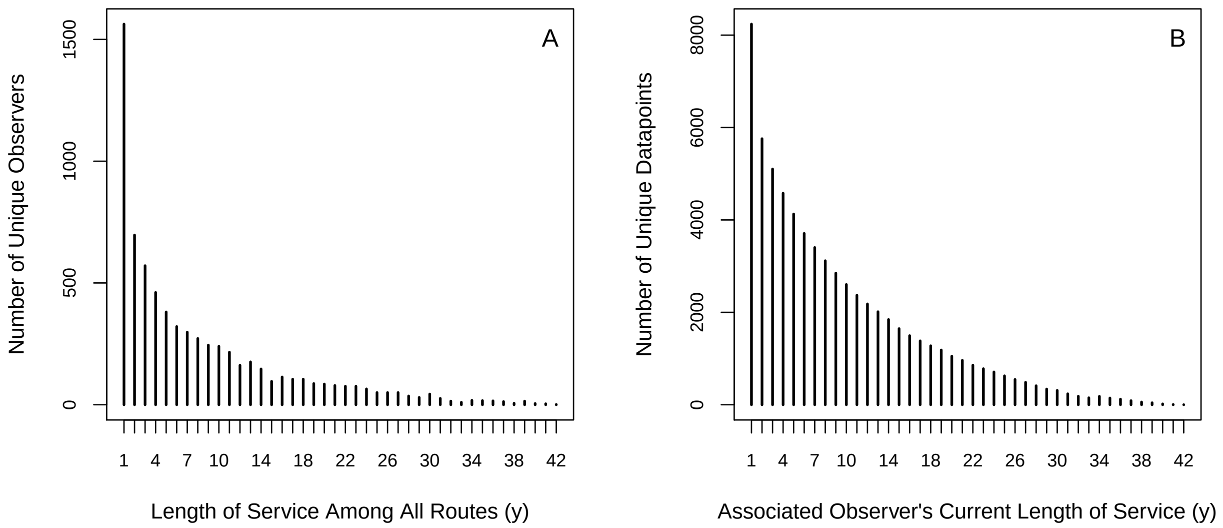

Figure S2. Measures of BBS observer age by (A) unique observer and (B) unique survey (unique combinations of observer, survey route and year), Canada and USA, 1966–2007. The distribution in Panel B is much less skewed towards short lengths of service than in Panel A.

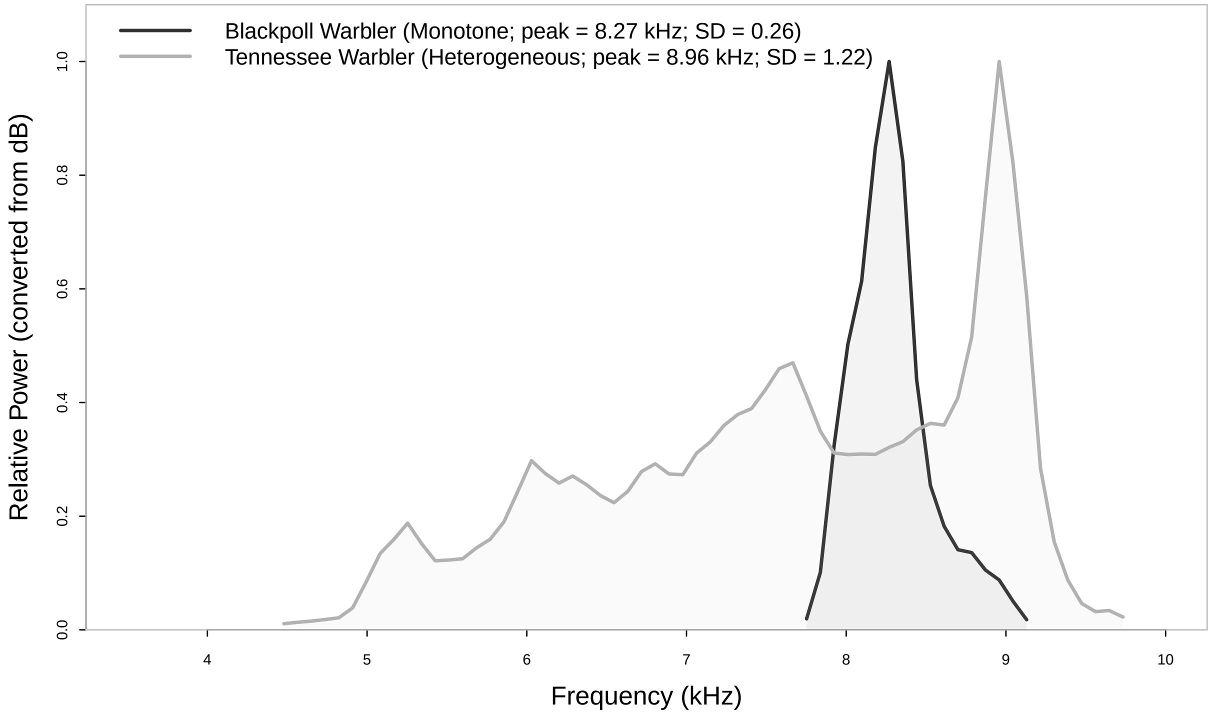

Figure S3. Examples of audiological power spectra corresponding to monotone (Blackpoll Warbler, *Dendroica striata*) and heterogeneous (Tennessee Warbler, *Oreothlypis peregrina*) vocalizations. The modified version displayed here presents the power as a linear-scale version of decibel values for each of a continuous range of frequency bins. Monotone vocalizations tend to feature a single or narrow range of frequencies, whereas heterogeneous vocalizations feature a wide range of sounds. Peak frequency values (kHz) and SD values (as a measure of heterogeneity) are listed for each species.

Appendix S2. Table of species used in the various hearing-loss analyses. 'OBBA' and 'BBS' refer to the analysis of raw data from the Atlas of the Breeding Birds of Ontario and the North American Breeding Bird Survey, respectively. 'USGS' and 'CWS' refer to analyses of population trends produced by the United States Geological Service and the Canadian Wildlife Service, respectively. Standard species abbreviations are taken from Klimkiewicz and Robbins (1978). Data describing a given species that were used for a given analysis are indicated by an asterisk in the corresponding row and column. Vocalization frequency information for each species, including peak vocalization frequency (Hz) and power spectrum standard deviation ('SD', as an index of call heterogeneity) are also provided. Frequency range and heterogeneity classifications are also provided, where *low* frequencies are less than 3 kHz, *'notch'* frequencies (corresponding to the audiometric notch related to noise-induced hearing loss) are between 3 kHz and less than 6 kHz, *medium* frequencies are between 6 and less than 7 kHz, and *high* frequency calls exceed 7 kHz. Heterogeneous vocalizations are in the upper 50% quantile of standard deviation values for a group of species that includes 19 additional, unmodeled species (not shown).

Species	Abbrev.	OBBA	BBS	USGS	CWS	Peak Freq. (Hz)	SD	Class
Red-breasted Nuthatch	RBNU	*	*	*	*	2670	514.22	Low Monotone
White-breasted Nuthatch	WBNU	*	*	*	*	2756	329.45	Low Monotone
Brown-crested Flycatcher	BCFL		*			2412	717.27	Low Heterogeneous
Great Crested Flycatcher	GCFL	*	*	*	*	2584	821.43	Low Heterogeneous
Ash-throated Flycatcher	ATFL		*			3101	319.57	Notch Monotone
Cassin's Kingbird	CAKI		*			3273	606.45	Notch Monotone

Continued on next page

Appendix S2, continued

Species	Abbrev.	OBBA	BBS	USGS	CWS	Peak Freq. (Hz)	SD	Class
Olive-sided Flycatcher	OSFL	*	*	*	*	3273	523.34	Notch Monotone
Western Wood-Pewee	WEWP		*	*	*	3445	405.60	Notch Monotone
Say's Phoebe	SAPH		*	*	*	3531	453.42	Notch Monotone
Scissor-tailed Flycatcher	STFL		*			3704	623.82	Notch Monotone
Gray Flycatcher	GRFL		*			3790	678.03	Notch Monotone
Pygmy Nuthatch	PYNU			*	*	3790	317.78	Notch Monotone
Grace's Warbler	GRWA		*			3876	489.64	Notch Monotone
Eastern Wood-Pewee	EAWP	*	*	*	*	4048	475.55	Notch Monotone
Vermilion Flycatcher	VEFL		*			4048	620.00	Notch Monotone
Yellow-bellied Flycatcher	YBFL	*		*	*	4134	646.14	Notch Monotone
Pine Warbler	PIWA	*	*	*	*	4221	532.83	Notch Monotone
Alder Flycatcher	ALFL	*	*		*	4307	646.83	Notch Monotone
Brown-headed Nuthatch	BHNU		*			4393	576.00	Notch Monotone
Common Yellowthroat	COYE	*	*	*	*	4565	593.58	Notch Monotone
Eastern Phoebe	EAPH	*	*	*	*	4823	432.01	Notch Monotone
Kentucky Warbler	KEWA		*			4910	684.07	Notch Monotone
Black-throated Gray Warbler	BTYW			*	*	5082	618.78	Notch Monotone
Orange-crowned Warbler	OCWA	*	*	*	*	5082	562.35	Notch Monotone

Continued on next page

Appendix S2, continued

Species	Abbrev.	OBBA	BBS	USGS	CWS	Peak Freq. (Hz)	SD	Class
Lucy's Warbler	LUWA		*			5512	630.58	Notch Monotone
Palm Warbler	PAWA	*		*	*	3618	840.98	Notch Heterogeneous
Western Kingbird	WEKI		*	*	*	3618	1004.12	Notch Heterogeneous
Willow Flycatcher	WIFL	*	*		*	3618	693.10	Notch Heterogeneous
Ruby-crowned Kinglet	RCKI	*	*	*	*	3790	1144.54	Notch Heterogeneous
Yellow-breasted Chat	YBCH		*	*	*	3876	1212.40	Notch Heterogeneous
Mourning Warbler	MOWA	*	*	*	*	3962	891.97	Notch Heterogeneous
Hooded Warbler	HOWA	*	*			4048	761.85	Notch Heterogeneous
Black-throated Blue Warbler	BTBW	*	*	*	*	4221	713.75	Notch Heterogeneous
Cerulean Warbler	CERW	*	*			4221	879.96	Notch Heterogeneous
Yellow-rumped Warbler	YRWA	*		*	*	4307	832.30	Notch Heterogeneous
Black-throated Green Warbler	BTNW	*		*	*	4393	905.87	Notch Heterogeneous
Louisiana Waterthrush	LOWA		*			4565	796.63	Notch Heterogeneous
Northern Waterthrush	NOWA	*	*	*	*	4565	1111.92	Notch Heterogeneous
Acadian Flycatcher	ACFL		*			4823	755.32	Notch Heterogeneous
Dusky Flycatcher	DUFL		*	*	*	4910	763.74	Notch Heterogeneous
Magnolia Warbler	MAWA	*	*	*	*	4910	1283.08	Notch Heterogeneous
Connecticut Warbler	CONW			*	*	4996	1100.18	Notch Heterogeneous

Continued on next page

Appendix S2, continued								
Species	Abbrev.	OBBA	BBS	USGS	CWS	Peak Freq. (Hz)	SD	Class
MacGillivray's Warbler	MGWA		*	*	*	4996	708.54	Notch Heterogeneous
Black Phoebe	BLPH		*			5082	951.26	Notch Heterogeneous
Virginia's Warbler	VIWA		*			5082	696.80	Notch Heterogeneous
Hermit Warbler	HEWA		*			5168	919.93	Notch Heterogeneous
Chestnut-sided Warbler	CSWA	*	*	*	*	5340	1101.86	Notch Heterogeneous
Prairie Warbler	PRWA		*			5340	855.07	Notch Heterogeneous
Yellow Warbler	YWAR	*	*	*	*	5340	889.48	Notch Heterogeneous
Townsend's Warbler	TOWA		*	*	*	5512	860.07	Notch Heterogeneous
Wilson's Warbler	WIWA	*		*	*	5771	1122.54	Notch Heterogeneous
Canada Warbler	CAWA	*	*	*	*	5857	830.75	Notch Heterogeneous
Hammond's Flycatcher	HAFL		*	*	*	5857	973.81	Notch Heterogeneous
Yellow-throated Warbler	YTWA		*			5857	732.75	Notch Heterogeneous
American Redstart	AMRE	*	*	*	*	5943	915.55	Notch Heterogeneous
Golden-winged Warbler	GWWA	*	*	*	*	6029	424.40	Medium Monotone
Worm-eating Warbler	WEWA		*			6546	514.35	Medium Monotone
Blue-winged Warbler	BWWA	*	*	*	*	6632	591.23	Medium Monotone
Black-and-white Warbler	BAWW	*	*	*	*	6718	663.48	Medium Monotone
Cedar Waxwing	CEDW	*	*	*	*	6891	314.65	Medium Monotone

Continued on next page

Appendix S2, continued								
Species	Abbrev.	OBBA	BBS	USGS	CWS	Peak Freq. (Hz)	SD	Class
Brown Creeper	BRCR	*	*	*	*	6977	679.02	Medium Monotone
Eastern Kingbird	EAKI	*	*	*	*	6202	1171.83	Medium Heterogeneous
Nashville Warbler	NAWA	*	*	*	*	6202	961.56	Medium Heterogeneous
Ovenbird	OVEN	*	*	*	*	6202	991.24	Medium Heterogeneous
Least Flycatcher	LEFL	*	*	*	*	6718	1276.71	Medium Heterogeneous
Northern Parula	NOPA	*	*	*	*	6891	786.68	Medium Heterogeneous
Golden-crowned Kinglet	GCKI	*	*	*	*	7235	680.00	High Monotone
Bay-breasted Warbler	BBWA	*	*	*	*	7321	490.08	High Monotone
Cape May Warbler	CMWA	*	*	*	*	7580	375.51	High Monotone
Blackpoll Warbler	BLPW	*		*	*	8269	257.59	High Monotone
Prothonotary Warbler	PROW		*			7494	1213.31	High Heterogeneous
Blackburnian Warbler	BLBW	*	*	*	*	7666	828.64	High Heterogeneous
Tennessee Warbler	TEWA	*	*	*	*	8958	1216.47	High Heterogeneous

Appendix S3. Vocalization heterogeneity

For each species, we obtained an audio recording of its typical vocalizations (calls and songs) from the Macaulay Library at the Cornell Laboratory of Ornithology (`http://macaulaylibrary.org`) and generated power spectra from each recording using the free software Audacity (Beta 1.3; `http://audacity.sourceforge.net/`). Power spectra display the total energy expended during an audio sample (dB) for each of a contiguous range of narrow frequency bins (i.e. 2.00–2.08 kHz, 2.081–2.160 kHz; Fig. S3 in Appendix S1). With this approach, the length of the recordings and the number of vocalizations featured in each recording were unimportant, as the power spectra considered the power and frequencies of all sounds present on each recording collectively.

By convention, sound intensities (power) are scored on the (logarithmic) decibel scale, which recognizes that human ears most readily distinguish changes in intensity along such an axis (Mayfield 1966). Converting a set of sound intensities to linear scales would tend to de-emphasize softer notes and highlight differences only among sounds of higher intensities. In our case, this linear-scale approach was appropriate for comparing vocalization variability because it tended to downplay any background noises present on a given audio track and emphasize only the dominant singing and calling notes of a given species. Accordingly, we first rescaled and linearized the log-scale decibel values within each power spectrum using the formula:

$$RelPower_i = 10^{(Power_i - Power_{max}) \cdot 0.1} \quad (1)$$

where $(Power_i - Power_{max})$ corresponds to the (negative) linear difference on the decibel scale between a given power value and the spectrum's maximum power value for $1, \ldots, i$ frequency bins. This function converts all decibel values to a scale from 0 to 1, where 1 equals the maximum power output, and it reflects linear-scale power differences (i.e. non-decibel values) between any given value and the maximum value.

For each set of transformed species vocalization data, we noted the peak acoustic frequency,

defined as the upper bound of the frequency bin with the highest power. We then treated the power spectra as histograms and determined the standard deviations of these 'distributions' to quantify their acoustic variability. We compared these standard deviation values among all species (including standard deviations from 19 additional, unmodeled species), and classified the vocalizations into 'monotone' and 'heterogeneous' groups. Heterogeneous vocalizations were in the upper 50% of standard deviation values (i.e. their calls were more variable); the remainder were classified as Monotone.

Appendix S4. Hierarchical occupancy model structure

The occupancy component of the models for each species was specified as:

$$z_i \sim Bernoulli(\psi_i) \tag{2}$$

$$logit(\psi_i) = A_0 + A_1 \cdot \zeta_i \tag{3}$$

for $i = 1, \ldots, 1212$ OBBA (atlas) squares, and where z_i corresponds to the unobserved true occupancy state of a given (second-atlas) atlas square (i.e. 0 or 1), $P(z_i = 1) = \psi_i$ (the occupancy probability for atlas square i), and ζ_i is a dummy variable indicating detection/nondetection (i.e. 0, 1) of a species by any observer in square i in the first atlas (1981–1985). A_0 and A_1 are logit-scale intercept and first-year occupancy parameters. Data used to determine ζ_i were derived from a set of 1,325 total observers from the first OBBA.

The detection component of the occupancy models for each species was specified as:

$$logit(p_{ij}) = \beta_1 \cdot \theta_{ij} + b_{obs_j} \tag{4}$$

$$b_{obs_j} = \beta_0 + \beta_2 \cdot Over50_j + \beta_3 \cdot Male_j + \epsilon_j \tag{5}$$

for $i = 1, \ldots, 1212$ atlas squares and $j = 1, \ldots, 350$ observers (or fewer, depending on the species being modeled), and where p_{ij} is the detection probability at square i for observer j, θ_{ij} is the natural log of effort, in party-hours, at square i by observer j, β_1 is the effort effect, and b_{obs_j} describes the observer effects. Among these observer effects (equation 5), β_0 is an intercept term, β_2 is the age (over-50 vs. under-40) effect, β_3 is the effect of being male, and ϵ_j is mean-zero, normally-distributed error about the observer effect, with the uniformly-distributed prior of the variance of this error having lower and upper bounds of 0 and 10, respectively. $Over50_j$ and $Male_j$ are dummy variables (0 or 1) indicating whether an observer is over age 50 (vs. under age 40), and whether that observer is male (vs. female).

The occupancy and detection models are combined in the overall hierarchy, which incorporates

observed detections Y_{ij}:

$$\mu_{ij} = z_i \cdot p_{ij} \tag{6}$$

$$Y_{ij} \sim Bin(N_{ij}, \mu_{ij}) \tag{7}$$

where Y_{ij}, the observed number of detections in square i for observer j is binomially distributed with probability of success μ_{ij} (the unconditional detection probability) for N_{ij} trials (i.e. the number of years during which an atlas square i was visited by observer j, which ranged from 2 to 5 detection-years).

Unless otherwise specified, all parameters in the hierarchical model (A_0, A_1, β_0, β_1, β_2, β_3) were assigned minimally-informative priors suitable for logistic regression models, which in most cases need not estimate absolute values greater than 5 (Gelman et al. 2008). We specifically used normally-distributed priors of standard deviation 3.16 ($\sqrt{10}$).

We used enough iterations in WinBUGS to achieve convergence of 3 Markov chains (with a burn-in of one half of the total), requiring that Gelman-Rubin Rhat statistics for all parameters be less than or equal to 1.1 to infer convergence. We also ensured that this model structure performed as intended by testing it with fake datasets of known observer characteristics (see electronic supplement for example code).

Appendix S5. Detailed methods for modeling changes in BBS count data with increasing observer age

To keep the more heavily-sampled species, observers or strata from having a disproportionate influence in our aggregated analysis, we modeled our BBS dataset over multiple stages using GAMMs. First, we modeled mean BBS counts for each species separately as overdispersed Poisson functions of both observer age and calendar year, correcting for differences among observers and survey routes as mean-zero, normally-distributed random intercepts. We used a cubic regression spline smooth term, chosen over thin-plate regression splines for computational efficiency reasons (Wood 2006), for each of the observer age and calendar year (i.e. population) effects, where the calendar year effects were smoothed separately for each stratum. The structure for each species-specific model was as follows:

$$\log(y_{i(j)kl}) = f_1(\tau_{kl}) + f_2(l)_j + \theta_k + \lambda_{i(j)k} + \sigma_{i(j)kl} \tag{8}$$

for $i = 1, \ldots, I$ routes within stratum $j = 1, \ldots, J$, $k = 1, \ldots, K$ observers, and $l = 1, \ldots, L$ calendar years since 1969, and where $y_{i(j)kl}$ is the number of birds detected on a route i in stratum j by observer k during year l, $f_1()$ and $f_2()_j$ are cubic spline smooth functions estimating age effects across the whole survey and population-related effects for physiographic stratum j, respectively, τ_{kl} is the (minimum) age of observer k in year l, θ_k are mean-zero, normally-distributed random intercepts for each observer, $\lambda_{i(j)k}$ are mean-zero, normally-distributed random intercepts for each observer at a route-within-stratum, $\sigma_{i(j)kl}$ is mean-zero, normally-distributed overdispersion error, and where datapoints collected by a given observer were weighted according to the inverse of the number of routes conducted by that observer for the modeled species.

To properly recognize the changes in BBS counts predicted by the smooth function $f_1()$ in these models (Equation 8), we did not simply extract its values for the modeled range of observer ages, since this approach would ignore the uncertainty among the separate

population-related smooth terms (estimated for each stratum; $f_2(l)_j$). Instead, working on the scale of the response variable, we defined species- and observer age-specific count predictions as the average of predictions for each relevant physiographic stratum. Calendar years were fixed at the midpoint of surveyed dates during predictions. We inferred the standard error about these averaged predictions, $\bar{\sigma}_{kl}$, as the square root of the mean of their variances.

We then built an 'aggregating' GAMM which generalized the predicted changes in BBS counts for each species with increasing observer age (produced above) among each of eight vocalization frequency groups (e.g. 'high monotone', 'notch heterogeneous'; discussed in Methods). In addition to generalizing the patterns of age-related count changes among species, this approach also ensured that each species contributed the same number of datapoints to the overall model. To convert the data to a common scale among all species, we used proportions of each species' maximum count as the (binomial) dependent variable in this model.

Similar to the single-species models (Equation 8), the aggregating GAMM used thin-plate regression spline smooth functions on observer age for each vocalization group, along with mean-zero, normally-distributed random intercepts for species. Each datapoint was weighted according to the inverse of its predicted coefficient of variation (i.e. $\frac{\hat{\mu}}{\hat{\sigma}}$). To provide a more useful interpretation of the species-independent changes in BBS counts with increasing observer age, final model predictions were then linearly rescaled relative to the values at observer-age 1 for each vocalization group. As in the detection probability analysis, we again ensured that this model structure performed as intended by testing it with fake datasets of known observer characteristics (see electronic supplement for example code).

References

Farmer, R. G., M. L. Leonard, and A. G. Horn. 2012. Observer effects and avian call count survey quality: rare-species biases and overconfidence. Auk 129:76–86, doi:10.1525/auk.2012.11129.

Gelman, A., A. Jakulin, M. G. Pittau, and Y. S. Su. 2008. A weakly informative default prior distribution for logistic and other regression models. Annals of Applied Statistics 2:1360–1383, doi:10.1214/08-AOAS191.

Kampstra, P. 2008. Beanplot: A boxplot alternative for visual comparison of distributions. Journal of Statistical Software 28:1–9.

Klimkiewicz, M. K., and C. S. Robbins. 1978. Standard abbreviations for common names of birds. North American Bird Bander 3:16–25.

Mayfield, H. 1966. Hearing loss and bird song. Living Bird 5:167–175.

Wood, S. N. 2006. Generalized Additive Models: An Introduction with R. Chapman and Hall, New York.
